# GPs opinions and perceptions of chiropractic in Sweden and Norway: a descriptive survey

**DOI:** 10.1186/2045-709X-21-29

**Published:** 2013-08-30

**Authors:** Daniel Westin, Tine Tandberg, Carol John, Iben Axén

**Affiliations:** 1 Anglo European College of Chiropractic, 13-15 Parkwood Road, Bournemouth, UK; 2 Elverum Kiropraktikk & Rehabilitering, Storgata 7B 2408, Elverum, Norway; 3 Karolinska Institutet, Institute for Environmental Medicine, Unit of Intervention and Implementation Research, Nobels v 13, 171 77, Stockholm, Sweden

**Keywords:** Chiropractic, Sweden, Norway, GP, Survey

## Abstract

**Background:**

In Sweden, chiropractic is not included in mainstream health care. In Norway chiropractic is a recognized health care profession. The aim of this study was to explore the perceptions of chiropractic among Swedish and Norwegian General Practitioners (GPs).

**Methods:**

Eight hundred surveys in each country were distributed randomly by post to Swedish and Norwegian GPs offices. The survey contained two main sections: Experiences and opinions about chiropractic and referral patterns. The data were then described and compared between the countries.

**Results:**

In Sweden the response rate was 44.8% and in Norway 45.3%. More than half of the Swedish GPs participating in this study stated that they had poor knowledge about chiropractic, while just a tenth of Norwegian GPs stated the same. Nearly all Norwegian GPs had some experience of chiropractic treatment whilst a fairly large number of the Swedish GPs said that they had no experience at all of chiropractic. It was twice as common for GPs in Norway to refer patients to a chiropractor as compared to Sweden. However, Swedish and Norwegian GPs agreed that chiropractors were competent to treat musculo-skeletal conditions with an adequate education to be part of mainstream medicine.

**Conclusions:**

Swedish and Norwegian GPs agree that chiropractors are competent to treat musculoskeletal conditions. However, there are many differences in GPs perceptions of chiropractic between the two countries and the overall picture indicates that chiropractic is more accepted and recognised as a health care profession in Norway.

## Background

There are approximately 550 chiropractors practising in Sweden [1,2]. Two hundred of these have qualified from colleges approved by the CCEI (the Council of Chiropractic Education International), and all are members of the Swedish Chiropractic Association (SCA). The remaining 350 chiropractors have received their training at the Scandinavian College of Chiropractic in Stockholm which does not meet the demands of the CCEI (through its European member, the European Council on Chiropractic Education) and therefore has no European accreditation [1]. However, all chiropractors have full legislation from the National Board of Health and Welfare in Sweden [2], regardless of education.

In Norway, there are approximately 400 chiropractors who are all members of the Norwegian Chiropractic Association, NCA. A membership in the NCA is granted only to graduates from CCEI accredited colleges [3].

Chiropractors in Sweden are not privileged to take x-rays, refer to medical specialists or to authorise sickness absence. There is generally no financial reimbursement from the Swedish government for patients seeking chiropractic care, even though local counties may have such agreements with chiropractors and other health care professionals [4]. Furthermore, chiropractic treatment for children was not allowed until recently [2]. The Swedish National Board of Health and Welfare [5] states in their guidelines that patients with chronic shoulder, neck and low back pain should be offered combination of different treatment methods including physiotherapy and Cognitive Behavioural Therapy (CBT).

In Norway chiropractors are authorised to take their own x-rays, refer to medical specialists and to refer for special imaging. In addition patients are given a greater range of treatment options in the management of musculo-skeletal problems. In 2001 the Norwegian Parliament launched a trial project; the Referral Project and lasted for two years. Nearly a million patients were involved in three Norwegian counties and the aim of the study was to improve patient satisfaction and cooperation within the health care services and to enhance socio-economic savings [6]. The participating chiropractors were given the following privileges:

•The right to issue sickness absence permits to their patients for up to 8 weeks.

•The right to refer to medical specialists when needed.

•The authority to refer for physical therapy and specialist radiological examinations.

•Patients no longer needed a GP referral in order to see a chiropractor with fee subsidisation.

The main result of the trial project was that the cost of sickness absence payments from the Labour and Welfare Service decreased by 2.4% in the three involved counties. Patients also reported that they were more satisfied with the opportunity to choose their own care [6].

As a result of the trial project new legislation was passed by the Norwegian Parliament concerning chiropractors and chiropractic treatment [7]. Hence, patients in Norway became entitled to financial compensation for up to 14 weeks per year for chiropractic treatment without GP referral. In addition chiropractors were given the right to administer sickness absence for up to 8 weeks and refer patients to medical specialists [8].

Thus, the differences regarding chiropractic care in these two neighbouring countries are substantial. Generally, General Practitioners (GPs) act as gatekeepers in the health care system and subsequently see many patients that may be suitable for chiropractic care. It is possible that their views of chiropractic influence the level of integration of chiropractic in mainstream health care as they may influence their patients’ perceptions during the GP consultation.

The aim of this study was to investigate and to compare the experiences and opinions of chiropractic among Swedish and Norwegian GPs and their referral patterns to chiropractic care.

## Methods

Design: A questionnaire survey. The survey is based on two Master-projects from the Anglo-European College of Chiropractic in England. In Sweden, the questionnaires were distributed in August of 2011 and in Norway in July of 2009.

The questionnaire was constructed on the basis of previous, similar surveys with the same purpose [9,10]. The questions were modified to be applicable to Swedish and Norwegian medical doctors. The questionnaire used in this study is found in Additional file 1.

There were 13 questions, all closed ended and divided into two main sections:

•Opinions and experiences about chiropractic

•Referral patterns

In the first part of the questionnaire the GPs general knowledge and opinions about chiropractors, educational standards and terminology was assessed, as well as the GP’s experience of chiropractic.

In the second part of the questionnaire the GPs were asked how often they referred to chiropractors and what kinds of conditions that were commonly referred.

Furthermore, questions were asked about correspondence from chiropractors to the GPs, reasons for not referring for chiropractic care and which care provider they commonly referred to if not a chiropractor.

In addition, some demographic information regarding the respondent (gender, age, and years in practice) was collected.

As this study was directed at clinicians with little time to spare it was important that the questions were short, closed ended and easy to answer. However, based on the experiences from the Norwegian part of the survey, the GPs in Sweden were given the option to give additional comments.

As no identifying personal information was collected, merely GPs opinions, the project was deemed exempt from a formal ethical permission procedure. The questionnaire was approved by both the Swedish and Norwegian Chiropractic Associations and as undergraduate projects at the Anglo-European College of Chiropractic.

In Sweden, the addresses to the GP offices were found on the public website of each county council. In Norway, the names and addresses to the GPs were provided through the public internet pages of the Norwegian Labour and Welfare Administration.

The number of questionnaires sent to the counties was calculated in order to give a response rate roughly proportionate to the population and the number of GPs inhabiting each county. There are a total of 21 counties in Sweden, and 19 counties in Norway. In Sweden, 30 questionnaires were sent out to each county; except for Stockholm, Västra Götaland and Skåne that received 105, 80 and 60 questionnaires respectively as these three counties are the most populated. In Norway, 41 questionnaires were sent out to each county, expect for Oslo that received 62 questionnaires according to the principle of proportion mentioned above. Thus, in each country, a total of 800 questionnaires were distributed. This accounts for 15% of the total GP population in Sweden and 20% in Norway.

In order to avoid selection bias the questionnaires were sent out to every other GP office in the order they appeared on the list from the county councils website until the quota of surveys for each county was filled. In Sweden the addresses were written by hand with the purpose of personalising the letter and thereby increasing the probability of the respondents completing the survey. In Norway the addresses were typed on the envelope.

Each envelope also contained a covering letter, found in Additional file 2, informing the GP’s of the purpose of the study, and assuring them of their anonymity and the confidentiality of the study. They were also informed that by returning the questionnaire they gave their consent to take part in the study. One stamped addressed envelope for returning the questionnaire was also included in the envelope.

## Results

### Response rate

In Sweden, 359 questionnaires with complete answers were returned. In addition, 12 questionnaires were returned with incomplete answers and were therefore excluded from the study. Another two letters were returned unopened due to retirement. Thus, the response rate of completed questionnaires was 44.8%. In Norway, 387 questionnaires were returned, of which 376 were complete. Ten questionnaires were returned due to incorrect addresses and 1 was returned due to retirement, rendering a response rate of 45.3%.

### Demographic data

The sample of Swedish GPs was slightly older than the Norwegian sample. Fifty-nine per cent of the Swedish GPs were aged 51 years or older whereas the corresponding number in Norway in this age group was 43%. Only one GP in Sweden was aged between 20 and 30, whereas in Norway, 19 GP’s fitted this category. Of the Swedish GPs, 58% were male and in the Norwegian sample 64% were male.

### Opinions and experiences of chiropractic

The questions and answers are summarized in Table 1.

**Table 1:** Summary of questions in the survey and the responses from the Swedish and Norwegian GPs

**Question and answer options**	**Sweden -%**	**Norway -%**
My knowledge of chiropractic		
- Good	7	22
- Know something	40	66
- Poor	53	12
- Never heard of it	0	0
My experience with chiropractic care		
- Good	47	66
- Bad	12	3
- None	21	3
- Other	20	28
I agree that		
- Chiropractic education is sufficient for mainstream medicine	49	80
- Chiropractors are competent for MSK conditions	69	93
- Chiropractors are competent to treat neurologic disturbances	25	17
- Chiropractors report poorly back to GPs	55	67
- Chiropractors use unknown terminology	11	25
I refer to a chiropractor	43	79
I refer the following conditions to a chiropractor		
- Acute back pain	37	68
- Chronic back pain	30	58
- Sports trauma	13	14
- Whiplash injuries	6	17
- Disc herniation	7	14
- Prolapse with uncomplicated neurological findings	16	24
- Migraine	2	8
- Tension headaches/headaches originating from the neck	9	9
- Asthma	0.5	1
- Carpal tunnel syndrome	0.5	1
- Back and pelvic problems during pregnancy	7	22
- Lateral/medial epicondylitis	6	7
- Nerve entrapment syndromes	7	5
- Infantile Colic	0	23
- Shoulder/knee pain	14	17
- Benign paroxysmal positional vertigo	0.5	5
- Other:	0	1
I refer MSK complaints to		
Chiropractors	31	82
Manual therapists	3	75
Physiotherapists	96	95
Osteopaths	1	7
Acupuncturists	12	15
Naprapaths	26	3
Homeopaths	0	0
I do not refer to a chiropractor because		
Poor knowledge about chiropractic treatment	38	16
They charge too much	26	14
Possible side-effects of chiropractic	14	12
Not sure how effective the treatment is	25	25
No chiropractors in my area	8	6
I have had bad experiences with chiropractors	4	8

The questionnaire started with the general question; “How would you describe your knowledge of chiropractic?

Seven per cent of the Swedish GPs felt that they had good knowledge of chiropractic and 53% felt that they had poor knowledge. The remaining 40% claimed to know something about chiropractic. In Norway, 22% of the GPs felt that they had good knowledge, 12% said that they had poor knowledge and 66% claimed to know something about chiropractic.

The GPs were asked if, in their opinion, chiropractors have a satisfactory education for mainstream medicine. Forty-nine per cent of the respondents in Sweden agreed and 42% had no opinion. In Norway, the corresponding figures were 80% (satisfactory education for mainstream medicine) and 18% (no opinion). Swedish and Norwegian GPs agreed that chiropractors were competent to treat musculo-skeletal conditions (69% and 93% respectively).

The GPs were asked if they thought that chiropractors use unusual terminology, and this was agreed by 11% of the GP’s in Sweden. In Norway 25% of the respondent GPs agreed.

The GPs were asked how they would describe their experiences of chiropractic treatment for their patients. Nearly half (47%) of the Swedish GPs and 66% of the GPs in Norway reported a good experience of chiropractic. Twenty per cent of the Swedish GPs reported to have a variable experience and 12% said that they had bad experience with chiropractic care for their patients. In Norway 27% of the GPs said that their experience was variable and 3% answered that they had a bad experience with chiropractic care. Furthermore, 21% of the GPs in Sweden stated that they have no experience of chiropractic. This figure in Norway was 3%.

### Referrals

In Sweden, GPs cannot refer patients to a chiropractor, so for the purpose of this study the questions about referrals in reality translate to a recommendation, rather than a referral for chiropractic care. The GPs were asked if they recommend chiropractic care for their patients and if so, how many times per month?

Forty-three per cent of the GPs in Sweden recommend their patients to see a chiropractor. In Norway, the majority, 79%, refer patients to a chiropractor. The reported rate of recommendation or referral was 1–5 times per month in both countries.

The GPs were asked what types of conditions they commonly refer to a chiropractor. Acute and chronic back pain followed by disc prolapse with uncomplicated neurological symptoms were the most common conditions to refer, for both Swedish and Norwegian GPs. However, more than 20% of the Norwegian GPs referred babies with colic to the chiropractor. This was non-existent in Sweden.

In response to the question about communication, 55% of the Swedish GPs and 67% of the Norwegian GP’s reported that there were poor levels of reporting back to the GPs from the chiropractors.

The common reasons for not referring amongst the Swedish GPs were; poor knowledge about chiropractic, not being sure of the effectiveness of chiropractic treatment and that it was too expensive for the patients. The reasons for not referring for chiropractic care in Norway were that GPs doubted the effectiveness of chiropractic care and that they had insufficient knowledge about chiropractic.

In Sweden GPs most often refer patients to physiotherapists followed by chiropractors and naprapaths. In Norway it was most common to refer patients to physiotherapists followed by chiropractors and manual therapists.

### Additional comments

There were twice as many negative comments about chiropractic as there were positive comments from the GPs in Sweden. The negative comments were concerned with chiropractors having different levels of education, over-treating patients and only focussing on short term relief. Also, some of the comments were concerned with the risks associated with chiropractic manipulation. Concerning positive comments, some of the GPs stated that they wanted increased cooperation with chiropractors and that they found manipulation beneficial, especially for low back pain. Five of these GPs also stated that they had great respect for the chiropractors that were trained at the Anglo-European College of Chiropractic.

## Discussion

In this questionnaire survey, the experiences and opinions of Swedish and Norwegian GPs about chiropractic were explored.

Just under half of the distributed questionnaires were returned, which is not unusual for studies of this nature [9]. As the responses were anonymous, it is not possible to perform an analysis to assess possible bias of this response rate. It is quite possible that the majority of the GPs (who did not return their questionnaires) had a totally different experience and opinion of chiropractic than what can be extracted from this study. However, the resulting picture sheds light of chiropractic’s position in the two countries, and seems to be informative regarding the matters under investigation. In Sweden, the survey was sent in handwritten envelopes. However, the response rates were almost identical in the two countries, leaving the importance of this additional effort subject to doubt.

As the survey is based on two Master-projects, they were distributed two years apart which may limit the comparison of the results between the countries. However, there were no changes in the legislation concerning chiropractic during these two years in either country, thus we do not believe that the time gap has any major role in the measured perceptions and opinions. Moreover, the experience of the first survey in Norway informed the space for additional comments in the Swedish version.

The Swedish sample of GPs was older than the GPs in the Norwegian sample. The importance of this finding on the results is unknown, but one may speculate that the older practitioners have a poorer knowledge of alternative and complementary medicine in general, which may be reflected in the results.

### Opinions and experiences about chiropractic

There are clear differences between the GPs in the two countries concerning their perceived knowledge about chiropractic, with the Swedish GPs reporting poorer knowledge than their Norwegian colleagues. The difference in the age distribution mentioned above may be responsible for this disparity. Perhaps the younger GPs are more aware of complementary medicine? There might also be educational differences between the younger and older GPs influencing the knowledge of chiropractic. An alternative explanation may be that in Sweden there are fewer chiropractors relative to the size of the population compared to Norway, which makes the Swedish GPs less exposed to chiropractic which could possibly be reflected in their lack of knowledge about the chiropractic profession.

About half of the GPs in Sweden thought that chiropractic education was satisfactory for mainstream medicine, and 80% of the Norwegian GPs thought so. As there are differences in the educational standards for chiropractors practising in Sweden, the profession is less homogenous which may lead to a perceived variation in the quality of care reflected in the lower Swedish score.

Chiropractic terminology and mainstream medicine terminology are different and this may potentially be problematic. However, very few (11%) of the Swedish GPs thought so, but 25% of the Norwegian GPs were aware of this potential problem. One explanation may be due to protocol differences between the countries when it comes to communication between GPs and chiropractors. In Sweden it is not mandatory for chiropractors to contact the GP after referral, whereas in Norway the chiropractor is obligated to contact the GP under such circumstances, Therefore, more communication is taking place between the chiropractors and the GPs in Norway compared to Sweden, which might explain the differences between the countries for this particular question.

In rating their experience of chiropractic, there were also marked differences between the countries. In Sweden, less than half of the GPs reported a good experience, and 21% stated that they had no experience of chiropractic care. The corresponding figures for the Norwegian GPs were two-thirds with good experience and only 3% had no experience. Again, this could reflect the before mentioned poorer exposure to chiropractic and the variable quality of chiropractic education in Sweden.

### Referrals

Chiropractic in Sweden is not part of mainstream medicine and GPs cannot refer patients for chiropractic care in the same way that they refer patients for physiotherapy as chiropractic is not included in the public health care system. Therefore it is challenging to compare referral patterns between the two countries. In Norway chiropractic is accepted as a viable option for patients with musculo-skeletal problems and is recommended by 79% of the GPs. This is almost twice the referral rate as in Sweden. Even if the Swedish GPs would like to refer patients to a chiropractor there are no official channels to do so and some GPs stated that they hesitate to refer patients because of the increased financial burden for the patient.

The conditions for which Swedish and Norwegian GPs refer patients to chiropractors are similar in the two countries. Acute and chronic low back pain with or without radiating leg pain were the conditions chiropractors were deemed competent to treat, which is consistent with the available evidence and for the scope of chiropractic care. The large difference in referrals for paediatric chiropractic care between the countries might be due to the fact that the ban on treating children in Sweden was only recently lifted, thus no tradition exists for this option. It may also be related to differences in recognition of the chiropractic profession in general.

There was agreement between the Swedish and Norwegian GPs that there were poor levels of reporting back to the GP. This pattern is seen in similar studies [9-11]. We speculate that an explanation for the poor levels of reporting back to the GPs might be differences in expectations of when a report is needed. Perhaps the GPs expect a report back for every patient they refer to the chiropractor and the chiropractor only reports to the GP when the patient’s condition change to a non- biomechanical condition that cannot be managed by the chiropractor. This is possibly an area where the chiropractic profession has room for improvement. If chiropractors reported back to GPs more frequently, the knowledge and understanding of chiropractic care would potentially improve.

Poor knowledge and not being certain of the effectiveness of chiropractic care were the most common reasons for GPs in both Sweden and Norway for not referring to a chiropractor. It is likely that poor knowledge and doubtfulness about the benefits of chiropractic care are influenced by each other. The growing evidence base of chiropractic care being effective in the treatment of acute and chronic low back pain, [12,13] was possibly reflected in the fact that these were the most common reasons for sending patients to the chiropractor. However, there may be other reasons responsible for the fact that many GPs doubt the effectiveness of chiropractic care. As mentioned in the comments, misconceptions about risks associated with chiropractic care, economic reasons and not being up to date with the latest evidence may all play a part. However, investigating these factors further was beyond the scope of this study.

Referring patients for physiotherapy was a very common option for the majority of GPs in both Sweden and Norway. This was an expected finding as physiotherapy has been within mainstream medicine for a long time and the patients in both countries receive financial subsidisation for this type of care.

The negative comments from the Swedish GPs may offer some insight into why the referral rates in Sweden are lower than in Norway. The GPs’ comments about different levels of education are justified because that is the case and a cause for concern in Sweden. To date, there are more chiropractors practicing in Sweden who are graduates from The Scandinavian College of Chiropractic than chiropractors from ECCE accredited colleges. Without a minimal level of academic quality in chiropractic education it might be difficult for the chiropractic profession to convince the medical profession that chiropractic is evidence based, effective and safe.

The comments concerning safety are linked to poor knowledge about the latest evidence. The most recent research suggests that there are very low risks associated with chiropractic manipulation [14].

## Conclusions

When comparing opinions, perceptions and experience of chiropractic among Swedish and Norwegian GPs there are clearly some differences.

More than half of the Swedish GPs participating in this study stated that they had poor knowledge about chiropractic, whilst the corresponding number in Norway was just 12%. A fairly large number of the Swedish GPs said that they had no experience at all of chiropractic but most Norwegian GPs had some experience of chiropractic treatment. It is also twice as common for GPs in Norway to refer patients to a chiropractor compared to Sweden.

There seems to be agreement among Swedish and Norwegian GPs that chiropractors are competent to treat musculoskeletal conditions. Further, most GPs think that chiropractic education meets the requirements of mainstream medicine. However the overall picture indicates that chiropractic is far more accepted and recognised as a health care profession in Norway. Lack of knowledge about chiropractic education, misconceptions about the risk of treatment as well as the lack of educational accreditation may explain these differences.

## Competing interests

The authors declare that they have no competing interests.

## Authors’ contributions

DW and TT adapted, distributed and analysed the surveys in Sweden (DW) and Norway (TT). CJ supervised the projects. IA was responsible for writing the manuscript together with DW. All authors read and approved the final manuscript.

## Supplementary Material

Additional file 1 The questionnaire. Click here for file

Additional file 2 Covering letter in Sweden. Click here for file
