# Supplementary material for: GPs opinions and perceptions of chiropractic in Sweden and Norway: a descriptive survey
Source: Chiropr Man Therap. 2013 Aug 30;21:29. doi: 10.1186/2045-709X-21-29 (PMC3765896; doi:10.1186/2045-709X-21-29)
Supplement: Additional file 1 — The questionnaire. [file 2045-709X-21-29-S1.docx]

**Additonal file 1 – The questionnaire**

**Personal information**

1.1 Age (in years): 20-30 31-40 41-50 51-60 >61

1.2 Gender: Male Female

1.3 When did you start working as a General Practitioner? …………………………………………

1.4 In which county do you practice medicine? …………………………………………………….

1.5 Is there a chiropractic practice present in your area? Yes No

**Experiences and Opinions Regarding Chiropractic and the Treatment given**

2.1 How would you describe your knowledge regarding chiropractic?

I consider my knowledge about chiropractic to be good

I know something about chiropractic

I have heard of chiropractic; however I consider my knowledge is poor

I have never heard of chiropractic

2.2 How would you describe your experiences with chiropractic treatment for your patients?

Good Bad No experience Other: ………………………………

2.3 In your opinion, which of these statements do you agree with?

Chiropractors: Agree Disagree Don’t Know

- Have a satisfactory education to be a part of mainstream medicine

- Are competent in the treatment of musculoskeletal complaints

- Are competent in the treatment of neurological disturbances

- Adequately report to the general practitioner on their findings

- Use unknown terminology in their report findings

**Referrals**

3.1 Do you refer patients to a chiropractor? Yes; go to 3.2

No; go to 3.4

3.2 Which of these complaints do you refer to a chiropractor? (You may tick one or more boxes)

Acute back pain

Chronic back pain

Sports trauma

Whiplash injuries

Disc herniation

Prolapse with uncomplicated neurological findings

Migraine

Tension headaches/headaches originating from the neck

Asthma

Carpal tunnel syndrome

Back and pelvic problems during pregnancy

Lateral/medial epicondylitis

Nerve entrapment syndromes

Infantile Colic

Shoulder/knee pain

Benign paroxysmal positional vertigo

Other: …………………………………………………………………………………………….

3.2 How often do you refer to a chiropractor?

1-5 times per month

6-10 times per month

11-15 times per month

16-20 times per month

More than 20 times per month: ……….. times per month

3.3 Who do you often refer your patients with musculoskeletal complaints to?

Chiropractor

Manual therapist

Physiotherapist

Osteopath

Acupuncturist

Naprapath

Homeopath

Other:………………………………………………………………………………………

3.4 What are your reasons for not referring to a chiropractor?

Don’t know enough about chiropractic treatment

They charge too much

Because of possible side-effects of chiropractic

Not sure how effective the treatment is

No chiropractors in my area

I have had bad experience with chiropractors

Other:…………………………………………………………………………………………

If you have any comments, please write them here:

Please send the questionnaire back in the pre-paid envelope.

Thank you for all your help.

Yours sincerely

Daniel Westin

4th year student at the Anglo European College of Chiropractic, Bournemouth.
