# Supplementary material for: GPs opinions and perceptions of chiropractic in Sweden and Norway: a descriptive survey
Source: Chiropr Man Therap. 2013 Aug 30;21:29. doi: 10.1186/2045-709X-21-29 (PMC3765896; doi:10.1186/2045-709X-21-29)
Supplement: Additional file 2 — Covering letter in Sweden. [file 2045-709X-21-29-S2.docx]

**Additional file 2 – The covering letter in Sweden**

Dear general practitioner,

I am a fourth year student at Anglo European College of Chiropractic, Bournemouth, England. I am interested in General Practitioner’s opinions and perceptions of chiropractic in Sweden as compared with Norway. I am hoping that my study may help to increase awareness between different health care providers in Sweden and potentially decrease the load on primary health care for musculoskeletal problems. The study has been approved by the Swedish Chiropractic Association.

The study involves completing a short questionnaire of 13 questions. The data will be completely anonymous; you have no obligation to take part and are free to withdraw from the study at any time. The data will be analysed and stored on a password protected database and used for the purpose of this study only.

Your involvement in this study is important and I would be very grateful for your participation. Please could you complete the survey and return in the pre-paid envelope. Your consent is given by completing this survey.

If you have any questions do not hesitate to contact me.

Thank you for your help.

Yours sincerely,

Daniel Westin

4^th^ year student at Anglo European College of Chiropractic, 13-15 Parkwood Road, Bournemouth, England.

westind@aecc.ac.uk
